# Zwitterionic Poly(sulfobetaine methacrylate) Brushes Functionalized Threads for DNA Extraction from Complex Cell Lysates

**DOI:** 10.3390/s25123651

**Published:** 2025-06-11

**Authors:** Xianlong Shi, Liang Wu, Ke Ning, Xinmei Li, Lingke Feng, Yirong Chen, Ling Yu

**Affiliations:** Key Laboratory of Luminescence Analysis and Molecular Sensing, Ministry of Education, Institute for Clean Energy and Advanced Materials, School of Materials and Energy, Southwest University, Chongqing 400715, China; shi1911324432@email.swu.edu.cn (X.S.); wuliang825@email.swu.edu.cn (L.W.); nk7868@email.swu.edu.cn (K.N.); mm0220@email.swu.edu.cn (X.L.); qiudoumadei@email.swu.edu.cn (L.F.); flourish@email.swu.edu.cn (Y.C.)

**Keywords:** SI-ATRP, PSBMA-functionalized thread, polymer brush, DNA extraction, solid-phase extraction, PCR

## Abstract

Thread-based analytical devices are low-cost, portable, and easy to use, making them ideal for detecting various biomolecules like glucose and DNA with minimal sample requirements, while also offering environmental benefits through their biodegradability. This study explores the potential of zwitterionic poly(sulfobetaine methacrylate) brushes modified cotton thread (PSBMA@threads) as an innovative substitute for DNA solid-phase extraction. The PSBMA polymer brushes were synthesized on cotton threads via surface-initiated atom transfer radical polymerization (SI-ATRP). The usability of the PSBMA@threads for DNA extraction from cell lysates containing cell debris, proteins, and detergents was evaluated. Characterization using SEM, FTIR, and EDS confirmed the successful functionalization with PSBMA polymer brushes. The antifouling properties of PSBMA@threads, including resistance to non-specific protein adsorption and underwater oil repellency, were assessed. The results demonstrated selective DNA capture from protein and lipid-rich lysates. Optimized extraction parameters improved DNA yield, enabling efficient extraction from tumor cells, which successfully underwent PCR amplification. Comparative experiments with commercial silica membrane-based columns revealed that PSBMA@threads exhibited comparable DNA extraction capability. The PSBMA@threads maintained extraction capability after six months of ambient storage, highlighting its stability and cost-effectiveness for nucleic acid isolation in analytical applications.

## 1. Introduction

DNA analysis plays a pivotal role in various fields, such as diagnostics (e.g., identifying circulating tumor DNA) [1], forensic odontology (e.g., criminal identification via DNA detection) [2], environmental monitoring [3], and biomedical research [4]. Accurate detection of DNA sequences enables the identification of pathogens such as viruses and bacteria [5], genetic mutations associated with diseases, and biomarkers for personalized medicine [6]. To ensure reliable results, the isolation of high-quality DNA from cellular lysates is a critical prerequisite for downstream analyses such as PCR, sequencing, and hybridization. Traditional DNA extraction involves two sequential steps: (1) cell lysis via physical (e.g., bead beating, sonication) or chemical (e.g., detergents, proteases) disruption to release nucleic acids, followed by (2) purification to isolate DNA from mixtures including proteins, lipids, and cellular debris [7,8,9,10]. Organic solvent-based methods such as phenol–chloroform extraction remain in use for DNA purification. However, these techniques might compromise DNA purity, utilize bio-hazardous reagents, and require multi-step centrifugation, rendering them impractical for point-of-care or high-throughput applications [11,12,13]. Solid-phase extraction (SPE) has emerged as a leading strategy for nucleic acid purification, facilitating selective interactions between DNA and functionalized surfaces [14,15]. For instance, DNA adsorbs onto silica membranes via chaotropic salt-mediated dehydration or onto cellulose-based matrices through hydrogen bonding, followed by low-salt elution [16,17,18]. However, conventional SPE materials face limitations such as high cost, and reliance on specialized equipment for fluid control [19]. Magnetic beads have become increasingly popular for DNA extraction due to their user-friendly handling and effective separation with magnetic field assistance. Surface modification and functionalization of magnetic beads can significantly enhance their capabilities in DNA extraction. For instance, silica-coated magnetic beads bind DNA effectively from complex biological mixtures. They also enhance extraction efficiency and purity, reduce non-specific binding, and minimize background interference. However, the surface modification and functionalization of magnetic beads are often costly and technically challenging processes. Additionally, magnetic beads can suffer from poor stability and limited reusability [20,21].

Thread-based analytical devices (μTADs) show great potential for low-cost, decentralized diagnostics. Their advantages include capillary-driven fluid transport, flexibility, and biocompatibility [22,23,24,25]. Compared to glass, silicon, and plastic materials, threads are more widely available, cost-effective, and easier to functionalize. These properties make them suitable for fabricating analytical devices, reducing the overall cost of biomolecular extraction and detection, particularly in point-of-care scenarios. Cotton threads offer a porous, hydrophilic structure that facilitates fluid transport and high surface-area-to-volume ratio, making them ideal for adsorption-based extraction. Recent studies demonstrate the utility of threads in immunoassays, metabolite detection, and nucleic acid extraction [26,27,28,29,30,31]. Their low cost, biodegradability, and ease of functionalization make them attractive alternatives to conventional SPE matrices for organic compounds isolation [32].

To extract DNA from cell lysate which contains proteins, lipids, and detergents, surface modifications of threads are required to enhance selectivity. Zwitterionic polymers, with their abundant charges and unique structures, promote DNA extraction (e.g., via zwitterionic polymer-coated magnetic beads for cell-free DNA) [33,34,35,36]. Among these, poly(sulfobetaine methacrylate) (PSBMA) stands out due to its antifouling properties and tunable electrostatic interactions [37,38,39,40,41,42,43,44,45,46]. In our prior work, PSBMA was grafted onto cotton threads via surface-initiated atom transfer radical polymerization (SI-ATRP), enhancing fluidic control, and enabling enzyme immobilization for biosensing applications [47].

In this study, we hypothesize that the zwitterionic PSBMA coating will minimize nonspecific adsorption of biomolecular contaminants while increasing DNA binding capacity of threads. The antifouling property of PSBMA coating could prevent protein non-specific adsorption on threads. While its underwater oleophobicity, which refers to a surface’s ability to repel oil droplets, helps inhibit the adsorption of lipid from cell membrane onto the threads. These dual actions facilitate DNA extraction from complex mixture. Consequently, PSBMA@threads can serve as an efficient and low-cost SPE matrix for DNA extraction. First, the PSBMA polymer brushes were synthesized on cotton threads via SI-ATRP approach. The functional group, and morphology of PSBMA@threads were examined by FTIR, and SEM analysis. The water absorption and wicking properties, protein repelling, and underwater oleophobicity of PSBMA@threads were compared with those of pristine cotton threads. The feasibility of DNA extraction was assessed by performing PCR on DNA isolated from varying cell quantities. The performance of PSBMA@threads was compared with that of conventional silica membrane-based nucleic acid purification columns. Furthermore, PSBMA@threads were stored at room temperature for six months, and their DNA extraction function were analyzed to evaluate stability.

## 2. Materials and Methods

### 2.1. Materials and Reagents

Cotton thread (1.5 mm diameter, 9-twist) was produced by Binzhou Yuchuang Chemical Fiber Rope Net Co., Ltd. (Binzhou, China). Cooking oil was from Yihai Kerry Arawana Holdings Co., Ltd. (Shanghai, China). Surface modification reagents including polyethylenimine (PEI), 3-glycidoxypropyl-trimethoxysilane (GPTMS), α-bromoisobutyryl bromide (BIBB), sulfobetaine methacrylate (SBMA), and ethylenediaminetetraacetic acid (EDTA) were from Sigma-Aldrich (Shanghai, China). Polymerization catalysts [CuBr, CuBr_2_, 2,2′-bipyridine, L-ascorbic acid] were sourced from Macklin (Shanghai, China).

Calf thymus DNA was purchased from Solarbio Co., Ltd. (Beijing, China). DNA purification kit and NA-Red 2000× were obtained from Beyotime Co., Ltd. (Shanghai, China). PCR components (Taq Mix, GAPDH, CDH1 primers), 25–500 bp, 50–500 bp, DNA marker were purchased from Sangon (Shanghai, China), Buffer RL was purchased from Vazyme Co., Ltd. (Nanjing, China). Oil red O, sodium hydroxide (NaOH), sodium carbonate (Na_2_CO_3_), and agarose were acquired from Aladdin Co., Ltd. (Shanghai, China). Silica membrane nucleic acid purification column was purchased from Doyou Biotechnology Co., Ltd. (Shanghai, China). Tris-HCl was purchased from Genview Co., Ltd. (Beijing, China).

Human prostate cancer cell DU 145 and murine breast cancer cell EMT-6 were purchased from Procell Life Science & Technology Co., Ltd. (Wuhan, China). The cells were cultured in Dulbecco’s Modified Eagle Medium (DMEM) supplemented with 10% fetal bovine serum (FBS, Bio-channel, Nanjing, China), penicillin (100 U/mL), and streptomycin (100 μg/mL) at 37 °C/5% CO_2_. Deionized (DI) water was used throughout the experiment (PURELAB flex System, ELGA Corporation, Lane End, UK).

### 2.2. Fabrication of PSBMA@thread

**Scheme 1:**
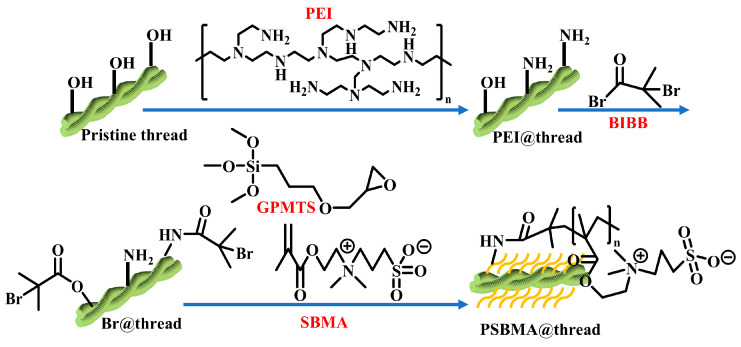
Illustration of the SI-ATRP grafting of PSBMA on cotton threads.

The PSBMA@threads were prepared according to the procedure illustrated in Scheme 1. In brief, cotton threads were treated in a boiling solution of NaOH (10 g/L) and Na_2_CO_3_ (10 g/L) for 30 min to remove surface wax. The threads were then washed with DI water and ultrasonicated for 30 min in 75% ethanol containing 1.0 mL GPTMS, 0.1 g PEI, and 2.5 mL acetate buffer. This step facilitated the reaction between GPTMS and threads, ensuring that the reaction solution uniformly coated the cotton threads. Then the reaction was followed by incubation in the dark for 24 h to ensure complete reaction of GPTMS, providing sufficient active sites for subsequent PEI grafting. The treated threads were dried at 100 °C for 1.5 h under vacuum condition to remove residual solvents and moisture. This step also involved fixing the spatial conformation of PEI molecules on the threads surface (PEI@threads). Next, PEI@threads were immersed in dichloromethane containing 0.52 g 4-dimethylaminopyridine and 4.3 mL triethylamine. After stirring under nitrogen for 30 min in an ice-water bath at 0 °C, 4.0 mL BIBB was added dropwise. The mixture was stirred in the ice-water bath for 1.5 h to minimize side reactions and ensure the efficient reaction between BIBB and the amino groups on PEI, generating bromine end groups (Br@threads). Then, the reaction continued at room temperature for 24 h to provide sufficient bromine end groups and initiation sites for subsequent SI-ATRP polymerization.

For the SI-ATRP grafting, 15 mg CuBr_2_, 36.2 mg 2,2′-bipyridine, and 2.4 g SBMA monomer were dissolved in 90 mL 50% (*v*/*v*) methanol/water solution. After vacuum treatment and nitrogen purging, 0.1 g ascorbic acid and 30 mg CuBr were added to initiate SI-ATRP at 60 °C for 24 h. For this CuBr/CuBr_2_ catalytic system, the reaction was kept at a mild temperature (60 °C) to initiate the controlled radical polymerization of SBMA monomers, generating a PSBMA brush layer. Finally, the PSBMA treated threads were soaked in 0.1 M EDTA overnight (solution replaced three times), washed with DI water, and dried at 60 °C to obtain PSBMA@threads.

### 2.3. The Morphology and Surface Properties of PSBMA@threads

Morphology and elemental composition were analyzed via SEM-EDS (Ultim Max 40, Oxford Instruments, Abingdon, UK). Before SEM measurement, the threads were sprayed with platinum for 240 s (JEC-3000FC, JEOL, Tokyo, Japan) to facilitate the SEM observation. The SEM operating parameters were as follows: the acceleration voltage was set at 10 kV, the beam current at 7 μA, and the working distance at 10 mm. Magnifications of 100×, 1000×, and 3000× were employed. Surface chemistry was assessed by FTIR (Spectrum Two™ FTIR Spectrometers, PerkinElmer, Waltham, MA, USA).

### 2.4. Characterization of the Function of PSBMA@threads

*Wicking rate*: Threads (5.0 cm) were horizontally suspended by attaching both ends to double-sided tapes. Then, 100 μL water was cast at one end of the thread. The average wicking rate (ν) was calculated using the formula ν = 5/*t*, where *t* is the time required for the water to completely wick through the thread.

*Water-absorbing property*: To measure the water-absorbing property, which reflects a thread’s affinity for water, dry threads were used in water-absorbing experiment. In brief, a 5.0 cm-long thread was soaked in water for 10 min, and the mass of the liquid-saturated thread was measured as m_0_. Then, the thread was dried in an oven at 65 °C for 3 h, after which its mass (m_1_) was measured. The difference between m_0_ and m_1_ indicated the amount of water adsorbed by threads.

**Scheme 2:**
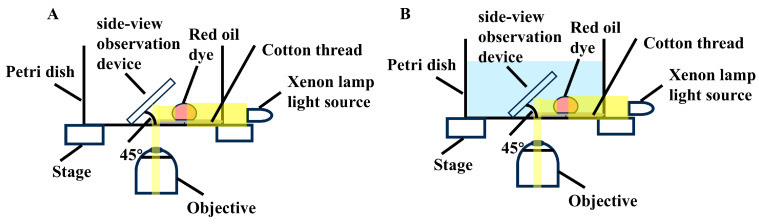
Illustration of side-view observation device (**A**). Threads were placed in a Petri dish and mounted on the sample stage of an inverted microscope. Red oil was applied to the threads (**B**). Water was added to immers the threads.

*Underwater Oleophobicity*: To assess underwater oleophobicity, a property referring to a surface’s ability to repel oil droplets, also known as non-adhesive superoleophobicity, 10 μL red oil dye (1 mg/μL) was added to the thread, and then water was added to submerge the thread. The leakage of oil drops from the thread underwater was recorded using a homemade side-view observation device [48]. The side-view device consists of a 3D-printed stand holding a first-surface-mirror at a 45° angle to the observing stage. During the measurement, the thread was placed in a Petri dish on the sample stage of an inverted microscope (TS100-F, Nikon, Tokyo, Japan). The mirror of the side-view observation device was positioned near the thread, less than 2.0 mm away (Scheme 2A). After depositing 10 μL of red oil dye onto the thread and capturing initial images, water was added to submerge the thread. The leakage of oil drops from the thread was then recorded by CCD camera of the microscope (Scheme 2B).

*Repelling protein adsorption*: To assess the ability of threads to resist protein adsorption, bovine serum albumin (BSA) was used as a model protein. BSA (10 μL, 1 mg/mL^−1^) was added on threads, and incubated for 30 min at 37 °C. Then the threads were washed with PBS and centrifuged at 5000 rpm for 3 min. Next, the residual protein on the threads were measured by a BCA assay kit (Ding guo Chang sheng, Beijing, China) according to the product instruction.

### 2.5. Optimization of PSBMA@thead-Based DNA Extraction Conditions

*The number of threads strand for DNA extraction*: 100 μL calf thymus DNA (662.26 ng/μL, measured using spectrophotometer, Model DS-11+, DeNovix, Wilmington, DE, USA) was incubated with threads with different twist numbers (1 cm each) for 10 min at 37 °C. Then, the DNA in the tubes was measured using spectrophotometer. The DNA extraction capacity of the threads was evaluated based on the difference in DNA concentration before and after incubation with the threads. Detailly, the extraction capacity was calculated using formula (C_0_ − C_1_), where C_0_ and C_1_ represent the DNA concentration before and after incubation with threads, respectively.

*Optimization of solid–liquid separation conditions*: 1 cm threads were immersed in solution and allowed saturating liquid. The solid–liquid separation was evaluated by measuring the mass of the threads before and after centrifugation. Centrifugation was performed at speeds ranging from 1000 to 9000 rpm, with durations of 1 to 3 min.

*Elution condition*: 30 μL calf thymus DNA solution (662.26 ng/μL) was added to threads and incubated for 10 min at 37 °C. Then centrifugation was performed to separate the solution and threads. Next, DNA-bound threads were washed with 75% ethanol, then eluted with diethylpyrocarbonate treatment water (DEPC water). To analyze the eluates by gel electrophoresis, 9 μL of the DNA sample was mixed with 1 μL of loading buffer by pipetting up and down to ensure thorough mixing. The mixture was then loaded into a 2% agarose gel. The gel electrophoresis was run at 120 V for 30 min. Then the gel was imaged using BOX Chemi XR5 Imaging System (Syngene, Cambridge, UK).

### 2.6. Thread-Based SPE to Extract DNA from Tumor Cells

**Scheme 3:**
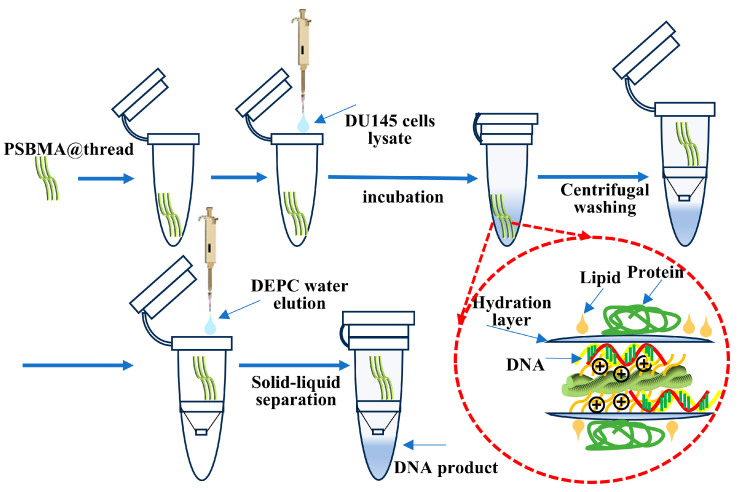
Scheme of extracting DNA from tumor cells via PSBMA@thread-based solid-phase extraction.

Human prostate cancer cell DU 145 and murine breast cancer cell EMT-6 were harvested for DNA extraction as illustrated in Scheme 3. A total of 1 × 10^5^ to 1 × 10^6^ cells were treated with 400 μL of cell lysis buffer (Buffer RL) for 2 min. The lysates were then loaded onto threads (1 cm × 3-twist) and incubated on a rocking plate shaker (KB-900, Beijing Mingchen Technology Inc., Beijing, China) for 10 min to facilitate DNA binding. The threads were centrifuged at 5000 rpm for 3 min to remove unbound material. The DNA was eluted from the threads using DEPC water. The extracted DNA was tested by PCR amplification of the GAPDH, and CDH1 genes as model. The PCR reaction consisted of a 50 μL total volume, containing 25 μL Taq Mix and 1 μM of each primer (forward and reverse). The primer sequences were shown in Table 1. The reaction mixture included 2 μL of the extracted DNA template, with the remaining volume made up with sterile and RNase/DNase-free water. The PCR was cycled 35 times under the following conditions: 95 °C for 15 s, Tm − 5 °C for 30 s, and 72 °C for 1 min. Finally, the PCR product was examined by DNA gel electrophoresis, as described above.

### 2.7. Statistic Analysis

The data were expressed as the mean ± standard deviation. Statistical analysis was performed using Student’s *t*-test (Origin Lab Corporation, Northampton, MA, USA) to assess the flow rate, water absorption, and protein repelling properties of pristine threads and PSBMA@threads. A *p*-value < 0.05 indicated statistical significance.

## 3. Results and Discussion

### 3.1. Characterization of PSBMA@threads

**Figure 1:**
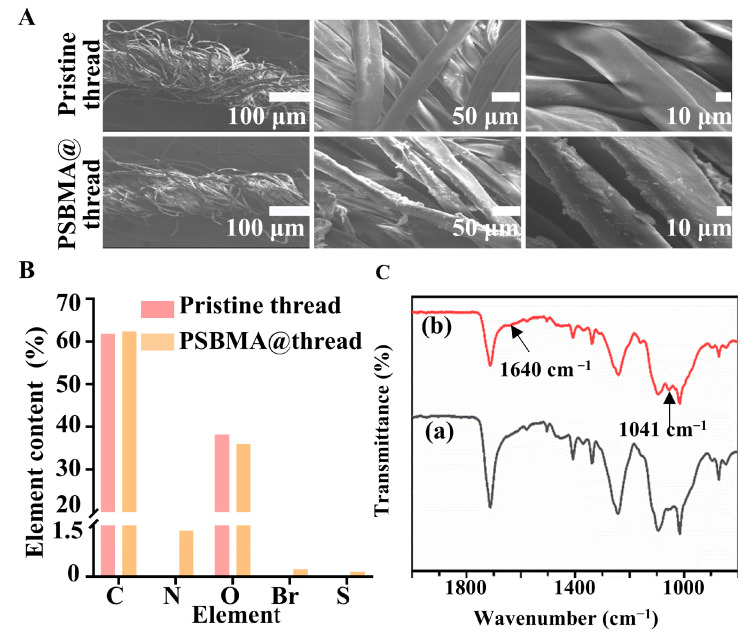
Characterization of threads. (**A**) SEM images of pristine threads and PSBMA@threads. **(B**) EDS quantified elemental content of threads. (**C**) FTIR Spectra of pristine threads (**a**) and PSBMA@threads (**b**).

*Morphology and function group of the threads*: As illustrated in Scheme 1, the sequential functionalization process involved grafting PEI and GPTMS onto threads to introduce amino groups. This enables nucleophilic substitution with BIBB, which initiated SI-ATRP of SBMA, yielding PSBMA@threads. SEM characterization revealed a rougher surface morphology for PSBMA@threads compared to pristine threads (Figure 1A), suggesting successful polymer brush grafting. EDS results of PSBMA@threads confirmed the presence of bromine (0.22%) and sulfur (0.15%), elements that were absent in pristine threads (Figure 1B). FTIR spectra showed a new peak at 1640 cm^−1^ corresponded to N–C=O stretching of brominated initiator. Additionally, the peak at 1041 cm^−1^ indicated the asymmetric stretching of the SO_3_^−^ group from SBMA (Figure 1C) [47]. The observed changes in surface morphology and the presence of characteristic elements and functional groups confirm the effective grafting of the PSBMA onto the thread surface.

**Figure 2:**
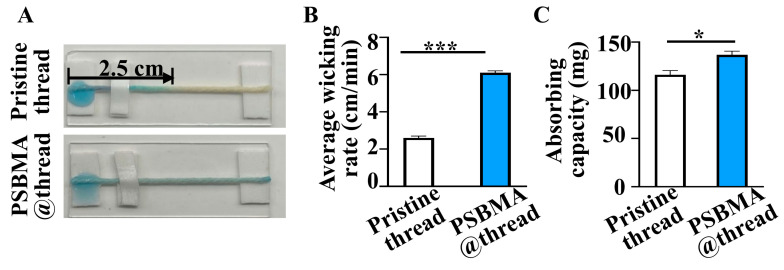
Flow characters of PSBMA@threads. (**A**) Comparison of liquid flow along a 5 cm length of pristine threads versus PSBMA@threads. Average wicking rate (**B**) and absorbing capacity (**C**) of pristine threads and PSBMA@threads (*n* = 3), * *p* < 0.05, *** *p* < 0.005.

*Water adsorption and flow characters of the threads*: Next, the liquid adsorption capability and flow characteristics of the PSBMA@threads were investigated. As shown in Figure 2A, water flowed through 5.0 cm PSBMA@threads, whereas the flow distance on pristine threads was only 2.5 ± 0.1 cm, indicating enhanced liquid transport. Quantitative analysis revealed that the pristine threads had a wicking rate of 2.6 ± 0.1 cm/min, which was significantly slower than the 6.1 ± 0.1 cm/min observed for PSBMA@threads (Figure 2B). Additionally, the water absorbed by pristine threads was 116.0 mg, compared to 136.8 mg for PSBMA@threads (Figure 2C). Thus, PSBMA@threads demonstrated a 15% higher water absorption capacity (136.8 ± 3.4 vs. 116.0 ± 3.9 mg, *p* < 0.05) relative to pristine threads. These improvements in liquid adsorption and wicking rate are attributed to the zwitterionic nature of PSBMA. The sulfonate groups enhance hydrophilicity through hydrogen bonding, while quaternary ammonium moieties facilitate electrostatic interactions (Scheme 1).

**Figure 3:**
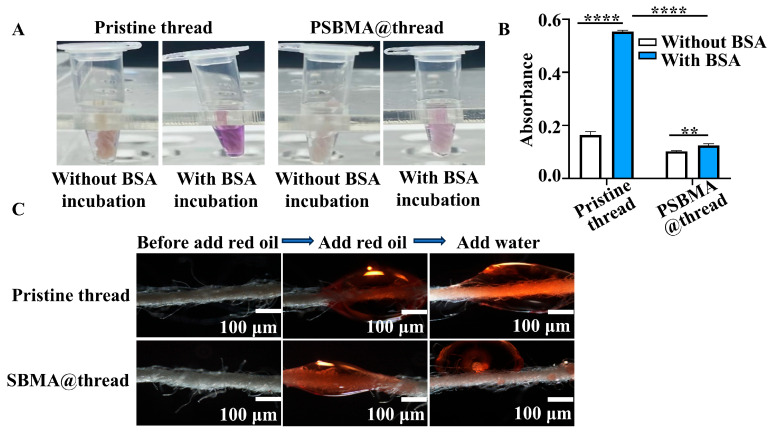
Protein repelling and underwater oleophobicity of PSBMA@threads. (**A**) Visual comparison of protein (BSA) adsorption resistance on pristine threads and PSBMA@threads using the BCA assay. (**B**) Quantitative analysis of protein absorbance on pristine threads and PSBMA@threads (*n* = 3) ** *p* < 0.01, **** *p* < 0.001. (**C**) Underwater oleophobicity of pristine threads and PSBMA@threads.

*Protein repelling and underwater oleophobicity*: Regarding non-specific protein adsorption, pristine threads exhibited greater protein adsorption than PSBMA@threads, as evidenced by a stronger purple color in the BCA protein assay (Figure 3A). Quantitative analysis via absorbance measurements at 570 nm confirmed significantly lower protein adsorption on PSBMA@threads (Figure 3B), demonstrating the effectiveness of the grafted PSBMA in repelling protein adsorption. Furthermore, when oil drops were added on the threads, they spread along the PSBMA@threads but quickly leaked away upon submersion in water (Figure 3C). This underwater oleophobicity, observed exclusively in PSBMA@threads, suggests that the zwitterionic polymer layer inhibits lipid adsorption. Given that cell lysates typically contain proteins, lipids, and other contaminants, the combined inhibition of non-specific protein and lipid adsorption by PSBMA@threads is expected to enhance DNA extraction capability from complex biological mixtures.

### 3.2. Optimization the PSBMA@threads-Based DNA Extraction

**Figure 4:**
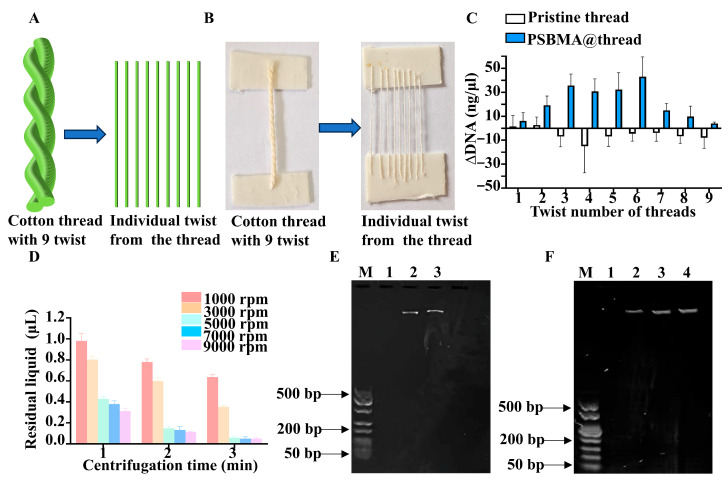
Optimization of the DNA extraction conditions. (**A**) Schematic of cotton threads with a 9-twist structure; (**B**) Pictures of thread with 9-twist and individual separated single-twist threads. (**C**) Optimization of the twist count in DNA extraction (*n* = 3). (**D**) Optimization of the centrifugation condition in solid–liquid separation (*n* = 3). (**E**) Optimization of the elution condition: lane 1. Negative control; lane 2. DNA eluted by sequentially added ethanol and DEPC water; lane 3. DNA eluted by DEPC water. (**F**) Optimization of the Eluent: lane 1. negative control; lane 2. DNA eluted by DEPC water; lane 3. DNA eluted by 0.1M NaCl; lane 4. DNA eluted by TE buffer (pH = 8.0).

The DNA extraction capacity of PSBMA@threads was evaluated using calf thymus DNA as a model analyte. A strand of cotton thread was composed of a nine-twist cotton thread as illustrated in Figure 4A. One strand thread was separated into nine individual twists to measure the DNA extraction potential (Figure 4B). The results showed that PSBMA@threads demonstrated more consistent extraction performance across a range of one- to nine-twist than that of pristine threads (Figure 4C). This suggests that, compared to pristine threads, the grafted zwitterionic polymer brush provides a balanced surface chemistry and physical structure for effective DNA binding. In addition, it was found DNA captured by a three-twist of PSBMA@threads was comparable to that captured by a four- to six-twist. Therefore, in the subsequent experiments, three-twist of PSBMA@threads were applied for DNA extraction.

Efficient solid–liquid separation is a crucial step in solid-phase extraction workflows to ensure high-purity DNA recovery. Centrifugation parameters were optimized to achieve complete separation of the PSBMA@threads from the surrounding liquid. Experimental data indicated that centrifugation at speeds of ≥5000 rpm for durations of ≥3 min consistently resulted in residual liquid volumes of less than 0.05 μL per thread (1 cm) (Figure 4D). Lower speeds or shorter durations led to incomplete separation, which could potentially contaminate downstream analyses. Therefore, for the subsequent DNA extraction experiment, centrifugation speed of 5000 rpm for a duration of 3 min were used.

A 75% ethanol solution was initially incorporated into the protocol as a washing solvent. Ethanol is commonly employed in nucleic acid purification to facilitate the aggregation and precipitation of DNA, which in turn enhances its retention on the solid support. However, as shown by the electrophoresis results in Figure 4E, ethanol did not enhance DNA recovery. Next, the impact of NaCl solution and TE buffer (Tris-EDTA, pH 8.0) was evaluated. The results revealed that the amount of DNA fragments eluted using standard DEPC water was comparable with that eluted with the 0.1M NaCl solution and TE buffer (Figure 4F). To simplify the experiment condition, in the following DNA extraction process, DEPC water alone was used as the elution buffer.

### 3.3. PSBMA@threads-Assisted Extraction of DNA from Tumor Cells for PCR Assay

**Figure 5:**
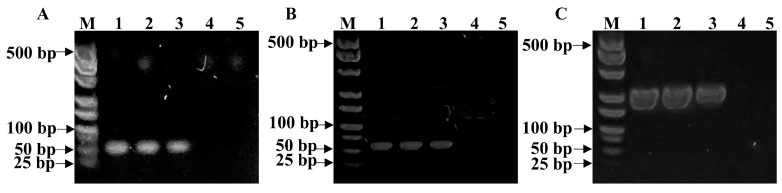
Characterization of PCR products using gel electrophoresis. (**A**) GAPDH gene from human prostate cancer cell DU 145 amplified from DNA extracted from 1 × 10^6^ DU 145 cells using PSBMA@threads (lane 1), 1 × 10^6^ DU 145 cells using commercial silica membrane nucleic acid purification column (ane 2), 1 × 10^5^ DU 145 cells using PSBMA@thread (lane 3), and 1 × 10^5^ DU 145 cells using a silica membrane nucleic acid purification column (lane 4). Lane 5 is negative control. (**B**) GAPDH gene from murine breast cancer cell EMT-6 amplified from DNA extracted from 1 × 10^6^ EMT-6 cells using PSBMA@threads (lane 1), 1 × 10^6^ EMT-6 cells using commercial silica membrane nucleic acid purification column (lane 2), 1 × 10^5^ EMT-6 cells using PSBMA@thread (lane 3), and 1 × 10^5^ EMT-6 cells using a silica membrane nucleic acid purification column (lane 4). Lane 5 is negative control. (**C**) Electrophoresis image of CDH1 gene amplified from DU 145 cells. The DNA templates used in each lane are the same as those in (**A**).

Following the optimized experimental conditions, the capability of PSBMA@threads for DNA extraction from tumor cells, DU 145 and EMT-6, was evaluated. The experiment utilized mixtures containing 1 × 10^5^–1 × 10^6^ tumor cells (Scheme 2). DNA extraction from these tumor cells was performed using PSBMA@threads and compared side-by-side with a commercial silica membrane nucleic acid purification column and pristine threads. The extracted DNA was then utilized for subsequent PCR experiments. The results demonstrated that PSBMA@threads could successfully extract DNA from 1 × 10^5^ cells, and this DNA was suitable for the expression analysis of target genes using PCR analysis. Glyceraldehyde 3-phosphate dehydrogenase (GAPDH) is a well-known housekeeping gene with functions in glycolysis [49]. GAPDH gene of human prostate cancer cell DU 145 and murine breast cancer cell EMT-6 were successfully amplified using the DNA template extracted by PSBMA@threads (Figure 5A,B). Apart from housekeeping genes like GAPDH, gene encodes functional protein was examined using the DNA extracted by PSBMA@threads. CDH1 gene encoding a protein called epithelial cadherin or E-cadherin, which is a calcium ion-dependent cell adhesion molecule that functions in the establishment and maintenance of epithelial cell morphology during embryogenesis and adulthood [50]. As shown in Figure 5C, CDH1 gene can be successfully amplified from DNA extracted by PSBMA@threads. However, the DNA extracted using commercial silica membrane purification column could only be used for PCR amplification from 1 × 10^6^ cells, but not 1 × 10^5^ cells (Figure 5). In addition, DNA extracted from pristine threads had a concentration of 2.4 ng/μL from 1 × 10^5^ DU 145 cells and failed in the PCR amplification experiment. The effectiveness of the grafted zwitterionic PSBMA polymer brush in repelling protein adsorption and exhibiting underwater oleophobicity facilitates the binding and extraction of DNA from cell lysates, which typically contain proteins, lipids, and other contaminants. This highlights the advantage of PSBMA@threads in extracting DNA from low-cell-number samples. Future work will explore the sensitivity limits and applicability of PSBMA@threads across diverse samples, such as plant cells, bacterial samples, and various cell lysis buffer compositions, including cetyltrimethylammonium bromide (CTAB)-based DNA extraction, to further validate their utility.

### 3.4. Stability of PSBMA@threads

**Figure 6:**
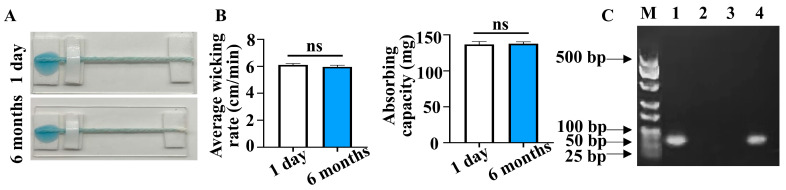
Stability of PSBMA@threads. (**A**) Comparison of liquid flow along a 5.0 cm length of PSBMA@threads stored for 1 day and 6-month. (**B**) Average wicking rate and absorbing capacity of PSBMA@threads stored for 1 day and 6-month (*n* = 3, ns: non-significant). (**C**) Agarose gel electrophoresis of GAPDH amplified from DNA extracted by different matrix, lane 1-six-month-stored PSBMA@threads, 2-pristine thread, 3-blank control, and 4-DNA extracted by commercial kit.

Assessing the long-term stability of PSBMA@threads is critical for their practical application. To evaluate this, the PSBMA@threads were stored under ambient conditions for 6 months. The capillary wicking rate and water absorption capacity were compared with freshly prepared PSBMA@threads. The results showed there was no statistical differences in either the wicking rate or water absorption between the two groups (*p* > 0.05) (Figure 6A,B), suggesting that the liquid transportation property of the PSBMA@threads remains stable over an extended storage period.

Subsequently, the DNA extraction capacity of three-twist number threads was tested after storage. The experimental results indicated no statistically significant difference in DNA extraction between PSBMA@threads stored for 1 day and those stored for 6 months (*p* > 0.05). Specifically, the DNA extraction capacity of three-twist PSBMA@threads stored for 1 day was 34.9 ± 3.7 ng/μL, while for those stored for 6 months, it was 25.7 ± 3.5 ng/μL. The decrease in DNA extraction capability lacked statistical significance (*n* = 3, *p* = 0.11). Finally, the six-month-stored PSBMA@threads were utilized to extract DNA from a DU 145 cell mixture. The extracted DNA was then subjected to PCR analysis to evaluate its quality and functionality. The results demonstrated that the DNA extracted using the six-month-stored PSBMA@threads maintained sufficient integrity and quantity to meet the requirements for PCR amplification (Figure 6C). This indicates that, despite a slight decrease in DNA extraction performance, the stored PSBMA@threads remain effective for nucleic acid extraction and subsequent analytical applications. Future work will explore the potential of storing extracted DNA on the threads for later elution and analysis. This approach could expand the utility of PSBMA@threads as a point-of-testing tool for on—site extraction, with the extracted DNA analyzed later in laboratories.

## 4. Conclusions

In this study, PSBMA@threads was prepared via SI-ATRP and their capability to extract DNA from DU 145 cells and EMT-6 cells for PCR application was evaluated. The results demonstrated that PSBMA@threads exhibited significantly improved liquid adsorption and antifouling properties compared to pristine cotton threads. The zwitterionic PSBMA brush layer minimized the adsorption of non-specific proteins and lipids while optimizing DNA capture. For the DNA extraction experiments, the centrifugation conditions were optimized to a speed of 5000 rpm for 3 min, and DEPC water alone was used as the elution buffer. PSBMA@threads were employed for DNA extraction from human prostate cancer cells (DU145) and murine breast cancer cells (EMT-6). The extracted DNA was successfully used for PCR amplification of the target genes GAPDH and CDH1.

Moreover, a comparison between PSBMA@threads and commercial silica membrane solid-phase extraction materials revealed that PSBMA@threads can extract DNA from 1 × 10^5^ tumor cells for PCR amplification of GAPDH and CDH1, whereas the silica membrane purification column could not. Additionally, PSBMA@threads retained efficient DNA extraction capabilities after six months of storage, demonstrating their excellent stability and cost-effectiveness. These findings suggest PSBMA@threads as a promising alternative for DNA extraction, with potential applications in molecular diagnostics and research, particularly in resource-limited settings.

## Figures and Tables

**Table 1:** Primer sequences.

**Cell line**	**Primer Name**		**Primer Sequence (5′→3′)**
DU 145	GAPDH	Forward	ctcctccgggtgatgctttt
		Reverse	gcccaatacgaccaaatcagag
	CDH1	Forward	agtcactgacaccaacgataat
		Reverse	atcgttgttcactggatttgtg
EMT-6	GAPDH	Forward	cttgtcatcaacgggaagcc
		Reverse	atgttagtggggtctcgctc

## Data Availability

The data in the current study are available from the corresponding authors upon reasonable request.

## References

[1] Gašperšič J., Videtič Paska A. (2020). Potential of modern circulating cell-free DNA diagnostic tools for detection of specific tumor cells in clinical practice. Biochem. Med..

[2] Sakari S.L., Jimson S., Masthan K.M., Jacobina J. (2015). Role of DNA profiling in forensic odontology. J. Pharm. Bioallied Sci..

[3] Bergman P.S., Schumer G., Blankenship S., Campbell E. (2016). Detection of Adult Green Sturgeon Using Environmental DNA Analysis. PLoS ONE.

[4] Mao F., Baiyin H., Li J., Chen X., Xu Y., Wang C., Li C. (2023). Editorial: Biomedical application of DNA modifications. Front. Genet..

[5] Charlermroj R., Makornwattana M., Phuengwas S., Meerak J., Pichpol D., Karoonuthaisiri N. (2019). DNA-based bead array technology for simultaneous identification of eleven foodborne pathogens in chicken meat. Food Control.

[6] Lai J., Du B., Wang Y., Wu R., Yu Z. (2018). Next-generation sequencing of circulating tumor DNA for detection of gene mutations in lung cancer: Implications for precision treatment. Onco Targets Ther..

[7] Scharf S., Bartels A., Kondakci M., Pfeffer K., Henrich B., Haas R. (2020). Introduction of a bead beating step improves fungal DNA extraction from selected patient specimens. Int. J. Med. Microbiol..

[8] Han Z., Sun J., Lv A., Sung Y., Sun X., Shi H., Hu X., Wang A., Xing K. (2018). A modified method for genomic DNA extraction from the fish intestinal microflora. AMB Express.

[9] Gómez-Acata E.S., Centeno C.M., Falcón L.I. (2019). Methods for extracting ‘omes from microbialites. J. Microbiol. Methods.

[10] Guo X.M., Gao W., Wang H.L., Wongkhaluang P., Taengchaiyaphum S., Xie G.S., Li C., Zhao R.H., Sritunyalucksana K., Huang J. (2024). Chitinase and proteinase K treatments enhance the DNA yield of microsporidium Ecytonucleospora hepatopenaei spores. J. Invertebr. Pathol..

[11] Barbier F.F., Chabikwa T.G., Ahsan M.U., Cook S.E., Powell R., Tanurdzic M., Beveridge C.A. (2019). A phenol/chloroform-free method to extract nucleic acids from recalcitrant, woody tropical species for gene expression and sequencing. Plant. Methods.

[12] Lutz Í., Miranda J., Santana P., Martins T., Ferreira C., Sampaio I., Vallinoto M., Gomes G.E. (2023). Quality analysis of genomic DNA and authentication of fisheries products based on distinct methods of DNA extraction. PLoS ONE.

[13] Barazesh A., Sarkari B., Ebrahimi S., Hami M. (2018). DNA extraction from hydatid cyst protoscolices: Comparison of five different methods. Vet. World.

[14] Llompart M., Celeiro M., García-Jares C., Dagnac T. (2019). Environmental applications of solid-phase microextraction. TrAC Trend Anal. Chem..

[15] Li P., Li M., Yue D., Chen H. (2021). Solid-phase extraction methods for nucleic acid separation. A review. J. Sep. Sci..

[16] Katevatis C., Fan A., Klapperich C.M. (2017). Low concentration DNA extraction and recovery using a silica solid phase. PLoS ONE.

[17] Ye X., Lei B. (2022). Improved DNA extraction on bamboo paper and cotton is tightly correlated with their crystallinity and hygroscopicity. PLoS ONE.

[18] Lindman B., Medronho B., Alves L., Norgren M., Nordenskiöld L. (2021). Hydrophobic interactions control the self-assembly of DNA and cellulose. Q. Rev. Biophys..

[19] Azizi A., Bottaro C.S. (2020). A critical review of molecularly imprinted polymers for the analysis of organic pollutants in environmental water samples. J. Chromatogr. A.

[20] Hou J., Hu C., Li H., Liu H., Xiang Y., Wu G., Li Y. (2025). Nanomaterial-based magnetic solid-phase extraction in pharmaceutical and biomedical analysis. J. Pharm. Biomed..

[21] Feng S., Zhang J., You J., Shi M., Yin L. (2025). Application of magnetic nanomaterials in sample pretreatment. Microchem. J..

[22] Min S., Zhan T., Lu Y., Pan D., Chen X., Xu B. (2023). Rapid and easily identifiable blood typing on microfluidic cotton thread-based analytical devices. Lab Chip.

[23] Oliveira A.C.M., Araújo D.A.G., Pradela-Filho L.A., Takeuchi R.M., Trindade M.A.G., Dos Santos A.L. (2022). Threads in tubing: An innovative approach towards improved electrochemical thread-based microfluidic devices. Lab Chip.

[24] Xiao G., He J., Chen X., Qiao Y., Wang F., Xia Q., Yu L., Lu Z. (2019). A wearable, cotton thread/paper-based microfluidic device coupled with smartphone for sweat glucose sensing. Cellulose.

[25] Tomimuro K., Tenda K., Ni Y., Hiruta Y., Merkx M., Citterio D. (2020). Thread-Based Bioluminescent Sensor for Detecting Multiple Antibodies in a Single Drop of Whole Blood. ACS Sens..

[26] Mao X., Du T.-E., Wang Y., Meng L. (2015). Disposable dry-reagent cotton thread-based point-of-care diagnosis devices for protein and nucleic acid test. Biosens. Bioelectron..

[27] Ulum M.F., Maylina L., Noviana D., Wicaksono D.H.B. (2016). EDTA-treated cotton-thread microfluidic device used for one-step whole blood plasma separation and assay. Lab Chip.

[28] Du T.-E., Wang Y., Zhang Y., Zhang T., Mao X. (2015). A novel adenosine-based molecular beacon probe for room temperature nucleic acid rapid detection in cotton thread device. Anal. Chim. Acta.

[29] Wu T., Xu T., Xu L.-P., Huang Y., Shi W., Wen Y., Zhang X. (2016). Superhydrophilic cotton thread with temperature-dependent pattern for sensitive nucleic acid detection. Biosens. Bioelectron..

[30] Li Y.D., Li W.Y., Chai H.H., Fang C., Kang Y.J., Li C.M., Yu L. (2018). Chitosan functionalization to prolong stable hydrophilicity of cotton thread for thread-based analytical device application. Cellulose.

[31] Chen L., Cabot J.M., Paull B. (2021). Thread-based isotachophoresis for DNA extraction and purification from biological samples. Lab Chip.

[32] Feng J., Han S., Ji X., Li C., Wang X., Tian Y., Sun M. (2019). A green extraction material-natural cotton fiber for in-tube solid-phase microextraction. J. Sep. Sci..

[33] Sun H., Zhou L., Chen X., Han X., Wang R., Liu H. (2016). Microscopic insight into the DNA condensation process of a zwitterion-functionalized polycation. Biopolymers.

[34] Nuckowski Ł., Dzieszkowski K., Rafiński Z., Studzińska S. (2021). Application of Magnetic Nanoparticles Coated with Crosslinked Zwitterionic Poly(ionic liquid)s for the Extraction of Oligonucleotides. Materials.

[35] He G., Wang W., Zhou Y., Zhao G., Liao J. (2024). Ampholytic ion-exchange magnetic beads: A promising tool for selecting short fragments in circulating cell-free DNA analysis. Front. Oncol..

[36] An M., Yesilbag Tonga G., Parkin S.R., Rotello V.M., DeRouchey J.E. (2017). Tuning DNA Condensation with Zwitterionic Polyamidoamine (zPAMAM) Dendrimers. Macromolecules.

[37] Sun H., Zhang Y., Li S., Bai Y., Ma J., Shao L. (2019). Multifunctional Core–Shell Zwitterionic Nanoparticles To Build Robust, Stable Antifouling Membranes via Magnetic-Controlled Surface Segregation. ACS Appl. Mater. Interfaces.

[38] Georgouvelas D., Jalvo B., Valencia L., Papawassiliou W., Pell A.J., Edlund U., Mathew A.P. (2020). Residual Lignin and Zwitterionic Polymer Grafts on Cellulose Nanocrystals for Antifouling and Antibacterial Applications. ACS Appl. Polym. Mater..

[39] Cheng Y., Wang J., Li M., Fu F., Zhao Y., Yu J. (2020). Zwitterionic Polymer-Grafted Superhydrophilic and Superoleophobic Silk Fabrics for Anti-Oil Applications. Macromol. Rapid Commun..

[40] Beltrán-Osuna Á.A., Ródenas-Rochina J., Gómez Ribelles J.L., Perilla J.E. (2018). Antifouling zwitterionic pSBMA-MSN particles for biomedical applications. Polym. Adv. Technol..

[41] Davenport D.M., Lee J., Elimelech M. (2017). Efficacy of antifouling modification of ultrafiltration membranes by grafting zwitterionic polymer brushes. Sep. Purif. Technol..

[42] Wang L., Sun L., Zhang X., Wang H., Song L., Luan S. (2022). A self-defense hierarchical antibacterial surface with inherent antifouling and bacteria-activated bactericidal properties for infection resistance. Biomater. Sci..

[43] Zhu L.-J., Zhu L.-P., Zhang P.-B., Zhu B.-K., Xu Y.-Y. (2016). Surface zwitterionicalization of poly(vinylidene fluoride) membranes from the entrapped reactive core–shell silica nanoparticles. J. Colloid Interf. Sci..

[44] Liu Y.L., Chang Y., Chang Y.H., Shih Y.J. (2010). Preparation of Amphiphilic Polymer-Functionalized Carbon Nanotubes for Low-Protein-Adsorption Surfaces and Protein-Resistant Membranes. ACS Appl. Mater. Interfaces.

[45] Liu C.H., Faria A.F., Ma J., Elimelech M. (2017). Mitigation of Biofilm Development on Thin-Film Composite Membranes Functionalized with Zwitterionic Polymers and Silver Nanoparticles. Environ. Sci. Technol..

[46] Liu Q.S., Patel A.A., Liu L.Y. (2014). Superhydrophilic and Underwater Superoleophobic Poly(sulfobetaine methacrylate)-Grafted Glass Fiber Filters for Oil-Water Separation. ACS Appl. Mater. Interfaces.

[47] Wu L., Xiong J., Xiao G., Ju J., Sun W., Wang W., Ma Y., Ran R., Qiao Y., Li C. (2024). Smart salt-responsive thread for highly sensitive microfluidic glucose detection in sweat. Lab Chip.

[48] Ning K., Fang C., Xie Y.Y., Chen Q.W., Feng L.K., Pan R., Yu L. (2025). A mirror-assisted imaging device enables side-view observation of microscale changes at interface without modifying the microscope. Measurement.

[49] Wang J., Yu X., Cao X., Tan L., Jia B., Chen R., Li J. (2023). GAPDH: A common housekeeping gene with an oncogenic role in pan-cancer. Comput. Struct. Biotechnol..

[50] Riethmacher D., Brinkmann V., Birchmeier C. (1995). A targeted mutation in the mouse e-cadherin gene results in defective preimplantation development. Proc. Natl. Acad. Sci. USA.

